# Green-Synthesized Nano-Silver Fluoride for Remineralization of Enamel Lesions in Primary Teeth: A Comparative In Vitro Study with SDF and SDF/KI

**DOI:** 10.3390/dj13070331

**Published:** 2025-07-21

**Authors:** Antonia Palankalieva, Plamen Katsarov, Ani Belcheva

**Affiliations:** 1 Department of Pediatric Dentistry, Faculty of Dental Medicine, Medical University of Plovdiv, 3 Hristo Botev Blvd, 4000 Plovdiv, Bulgaria; ani.belcheva@mu-plovdiv.bg; 2 Department of Pharmaceutical Technology and Biopharmacy, Faculty of Pharmacy, Medical University of Plovdiv, 15A Vassil Aprilov Blvd, 4002 Plovdiv, Bulgaria; plamen.katsarov@mu-plovdiv.bg; 3 Research Institute at Medical University of Plovdiv (RIMU), 4002 Plovdiv, Bulgaria

**Keywords:** nano-silver fluoride, green synthesis, silver diamine fluoride, enamel remineralization, dentistry

## Abstract

**Background**: Early caries management is essential to enable reversal of white spot lesions without the further need for operative interventions, especially in primary dentition. Silver-based compounds can be quite effective in arresting caries lesions; however, a major drawback is teeth staining. This study aimed to evaluate the remineralization potential and aesthetic effects of novel, green-synthesized nano-silver fluoride (NSF) on artificial white spot lesions in primary teeth in comparison to 38% silver diamine fluoride (SDF) and silver diamine fluoride/potassium iodide (SDF/KI). **Materials and Methods**: NSF was synthesized using green tea extract. Sixty primary teeth specimens with artificial enamel lesions were randomly divided into five groups depending on the applied material: 38% SDF, 38% SDF/KI, single and double application of NSF, and control group. Treatments were followed by pH cycling. Surface microhardness and photographic analysis were conducted to assess remineralization and staining. Statistical analysis was conducted using non-parametric tests including Kruskal–Wallis and Mann–Whitney U tests with Bonferroni correction. **Results**: The greatest increase in microhardness was observed in the group receiving double NSF application. Its remineralizing potential was comparable to that of 38% SDF/KI, with no statistically significant difference (*p* = 1.000). Importantly, NSF-treated samples exhibited no teeth discoloration, unlike the black staining observed in SDF and SDF/KI groups. **Conclusions**: Green-synthesized NSF is a promising alternative to conventional SDF and SDF/KI, offering remineralization benefits without compromising aesthetics. The eco-friendly formulation and non-staining properties support its potential for clinical use in pediatric dentistry.

## 1. Introduction

Early childhood caries (ECC) is a serious public health concern, as it is considered a chronic condition [1] with little to no improvement in the last 25 years [2]. Its treatment must be timely, effective, and accessible and ensure quality of life and comfort for pediatric patients.

Early caries management is essential to enable reversal of white spot lesions without the further need for operative interventions. The “golden standard” in the face of fluoride therapy has proven its benefits; however, new approaches for remineralization therapy are being invented and studied [3].

Historically, silver (Ag+) was used since ancient times [4]. Nowadays, its powerful antimicrobial action is very well established [5]. Numerous in vitro and in vivo research studies have proven that silver compounds have the potential to arrest and even prevent carious lesions in both deciduous and permanent teeth [6].

Currently, silver diamine fluoride (SDF) is the main choice when using silver products for caries management. Three main properties define the role of SDF in arresting caries progression—remineralization, antiproteolytic, and antibacterial properties [7].

The clinical application of SDF for dentine caries arrest is supported by several clinical studies [8,9,10,11,12,13]. Regarding the effect on enamel carious lesions, literature provides mainly in vitro studies [14,15,16]. The biggest disadvantage of SDF treatment has been the black staining of teeth, which has limited its use [17]. Certain approaches have been adapted to minimize the color change following SDF treatment, some of which include applying potassium iodide (KI) [18]. When KI is applied after SDF, this leads to white precipitate formation of silver iodide, and the spare silver ions that cause the staining are removed [19]. However, recently, it was observed that the improvement of the color after KI application was only temporary, and after some time, discoloration of the treated teeth still appears [20].

Nanoscience has gained a lot of attention in recent years in the field of dental research [21]. Some of the most popular objects of the studies in this field are silver nanoparticles [22]. They have been proven as potential antibacterial agents for dental decay prevention in children [23]. They are also capable of penetrating and disrupting the biofilm structure, making them suitable candidates for both prevention and management of one of the most prevalent oral health issues—ECC [24]. By combining fluoride ions and silver nanoparticles, a very effective formula has been created, which has both antibacterial and remineralization properties; therefore, it could be used for arresting white spot lesions in primary teeth [25]. Additionally, due to the nanostructure, no tooth staining has been caused [26]. Various techniques are used for nanoparticles synthesis, from which green methods stand out as very simple, cost-effective, and ecological [27]. The green synthesis of nanoparticles consist of using different plants, bacteria, fungi, and algae as reducing and stabilizing agents [28]. Among the various green sources for nanoparticles production, plant-based approaches are most commonly used [29]. A notable example is green tea, one of the most favored beverages worldwide, derived from the leaves of *Camellia sinensis* [30]. It mainly contains epigallocatechin, epigallocatechin-3-gallate (EGCG), epicatechin, and epicatechin-3-gallate, which serve as reducing and capping agents [31]. These polyphenols have exhibited significant antioxidant, anticarcinogenic, anti-inflammatory, probiotic, and antimicrobial properties in numerous human, animal, and in vitro studies [32]. Therefore, silver nanoparticles synthesized with green tea extract offer a promising strategy for managing dental caries in primary dentition [33].

The aim of the present study is to develop a novel, green-synthesized nano-silver fluoride (NSF) and test its efficacy in remineralizing artificial white spot lesions in primary teeth, in comparison with commercially available SDF and SDF/KI in vitro. In addition, the study investigates potential color changes in the dental tissues following application.

The null hypothesis states that there is no difference in enamel remineralization and staining between the deciduous teeth treated with NSF, SDF, and SDF/KI.

## 2. Materials and Methods

### 2.1. Formulation and Characterization of Green-Synthesized NSF, Structural and Morphological Analysis

NSF was synthesized via a green method using an aqueous extract of *Camellia sinensis* (green tea) leaves. Briefly, 1.5 g of ground dry leaves were boiled in 100 mL of purified water at 80 °C for 30 min, cooled, and filtered. The extract was diluted to 300 mL, and 50 mL of silver nitrate solution were added under constant stirring (800 rpm) at 80 °C. The pH was adjusted to 9 with potassium carbonate. The formation of nanoparticles was confirmed visually by a color change from yellow to dark brown. Sodium fluoride (0.01 g/mL) was then added. The final volume was reduced to 50 mL using a rotary evaporator at 50 °C (Rotavapor RII, Büchi, Flawil, Switzerland).

The synthesis was confirmed by UV-Vis spectroscopy (Evolution 300, Thermo Fisher Scientific, Waltham, MA, USA), scanning in the 200–600 nm range. The nanoparticle suspension was freeze-dried (Alpha 1-2 LSCbasic, Martin Christ, Osterode, Germany) at −55 °C, 0.1 mbar for 24 h, followed by final drying at 0.05 mbar for 2 h.

Structural and morphological analysis was performed via transmission electron microscopy (TEM; Talos F200C G2, Thermo Fisher Scientific, Waltham, MA, USA), dynamic light scattering (DLS; Nanotrac, Microtrac, York, PA, USA), and scanning electron microscopy (SEM; PrismaTM E, Thermo Fisher Scientific, Waltham, MA, USA) equipped with energy-dispersive X-ray (EDX) spectroscopy for elemental analysis.

### 2.2. Teeth Sample Preparation

Forty primary teeth (incisors, canines, and molars) with intact surfaces were collected at the time of their physiological exfoliation. Ethical approval was obtained from the Medical University of Plovdiv, Bulgaria (№ P-841, 08/04/2024), and informed consent was received from the guardians of all participants from whom teeth were collected. Teeth were cleaned with a polishing brush (Pro-Brush, Kerr, Kloten, Switzerland) and non-fluoridated paste (Cleanpolish, Kerr, Kloten, Switzerland), then sectioned 1 mm below the cementoenamel junction using a double-sided diamond disc (SuperFlex, SS White, Lakewood, NJ, USA) under water cooling. The primary incisors and canines (n = 20) were embedded whole in cylindrical blocks of fast-curing resin with a diameter of 12 mm (BMS Dental, Capannoli, Italy), with the vestibular surface facing upwards and parallel to the horizontal plane. The primary molars (n = 20) were sectioned longitudinally in a medio-distal direction, yielding two experimental halves (vestibular and lingual), resulting in 40 molar samples (n = 40). Each half of the separated primary molars was also embedded in resin blocks. On the resulting 60 samples, an experiment window of approximately 4 mm by 4 mm was created by covering half of their surfaces with two layers of acid-resistant nail polish (X-Treme Top Coat, Eveline Cosmetics, Lesznowola, Poland).

### 2.3. Induction of Artificial Initial Carious Lesions

To create initial carious lesions on the experimental specimens, a protocol adapted from Huang et al. [34] was used. Each specimen was individually immersed in a sterile glass container with 10 mL of demineralization solution for a period of 72 h at 37 °C. The solution was renewed with a fresh one every 24 h. After demineralization, the specimens were rinsed with distilled water and allowed to dry at room temperature.

### 2.4. Experimental Grouping and Treatment Protocol

The primary endpoint of this in vitro study was the change in surface microhardness after treatment with different silver-based agents.

Teeth samples (n = 60) were assigned to five groups (n = 12 per group) based on the applied treatment:

- Gr-SDF—Single application of 38% SDF (Riva Star Step 1, SDI Dental Limited, Melbourne, Victoria, Australia).
- Gr-RS—Single application with a two-step Riva Star capsule system (Riva Star Step 1 and Step 2, SDI Dental Limited, Melbourne, Victoria, Australia).
- Gr-NSFs—Single application of laboratory-synthesized NSF using a green method with green tea extract.
- Gr-NSFd—Double application of laboratory synthesized NSF using a green method with green tea extract at one-week interval.
- Gr-C—Control group with no treatment.

The required sample size was determined through a sensitivity analysis with G*Power software (version 3.1.9.7, Heinrich-Heine University, Düsseldorf, Germany). A one-way ANOVA (fixed effects, omnibus) was selected with five independent groups. The significance level was set at α = 0.05, and the desired statistical power was 95% (1–β = 0.95). With a total of 60 primary teeth samples (n = 12 per group), the analysis indicated that the study could detect an effect size of Cohen’s f = 0.58. According to Cohen’s criteria, this corresponds to a very large effect, indicating that the study design can detect strong and meaningful differences in surface microhardness between treatment groups under controlled in vitro conditions [35,36].

Allocation was performed using a computer-generated simple permuted block sequence to ensure randomness and balance of the groups.

Description of the silver materials used in this study and their application protocol are represented in Table 1.

Following treatment, the specimens were subjected to a 7-day pH cycling model adapted from Amaechi et al. [37], simulating the daily acid attacks to which teeth are exposed in the oral cavity. The pH cycling regimen for each day consisted of three one-hour acid challenges followed by storage in artificial saliva for 2 h. Compositions of the de- and remineralizing solutions are presented in Table 2. The same demineralizing solution was used for artificial caries induction and for the pH cycling protocol.

### 2.5. Surface Microhardness Evaluation

To assess the primary outcome, surface microhardness (Vicker’s hardness number—VHN) was measured at three stages: baseline (VHN-S), after demineralization (VHN-C), and after treatment and pH cycling (VHN-T). Measurements were taken using Vicker’s microhardness tester (Wilson Tukon 1102, Buehler Ltd., Lake Bluff, IL, USA) with a load size of 100 g, applied for 10 s. For each sample, three indentations were made 100 µm apart, and the calculated average value was used for analysis.

To minimize bias, all specimens were assigned randomly generated codes by a researcher not involved in outcome assessment. Microhardness testing was performed by a blinded examiner. However, due to the characteristic black staining, observed in some groups, complete blinding of the evaluator could not be fully maintained.

### 2.6. Photographic Analysis

To monitor the change in the samples color, all studied groups were photographed before and after the creation of the artificial carious lesions, on days 1, 3, and 7 after treatment with the respective silver-based product. The groups that received two-time treatment were photographed after the second application of the material. Standardized photographs were taken by an independent examiner who was not involved in the evaluation process, using a Canon EOS 50D digital camera, 2.8 Macro USM Lens, and a Macro Ring Lite Flash MR-14EX II (Canon, Tokyo, Japan) at a fixed distance of 40 cm against a white background under consistent lighting. The examiner was blinded to the treatment groups during image capture, although full blinding was not possible due to the observed black discoloration in some groups.

Prior to photographic analysis, two evaluators were calibrated using a separate set of images that were not included in the final study. This was done to ensure consistency in identifying discoloration. After calibration, each observer independently assessed all study images for the presence or absence of visible staining.

### 2.7. Statistical Analysis

For data processing and graphical presentation, the programs IBM SPSS Statistics for Windows (SPSS Windows, version 30.0, 2024, Chicago, IL, USA) and GraphPad Prism (GraphPad Prism, version 10.4.1, 2024 San Diego, CA, USA) were used.

Since the microhardness values of a healthy enamel surface vary widely, to facilitate comparison between the studied groups, two new variables were defined to assess the dynamics of changes in VHN:

- VHN-1: Difference between VHN after demineralization and baseline (VHN-C minus VHN-S), representing mineral loss.
- VHN-2: Difference between post-treatment and post-demineralization values (VHN-T minus VHN-C), representing remineralization.

The normality of the distribution was evaluated using the Shapiro–Wilk test. Differences among groups were analyzed using the Kruskal–Wallis test, followed by pairwise comparisons with the Mann–Whitney U test. The overall level of statistical significance was set at *p* < 0.05. Once these statistical tests were carried out, Bonferroni correction was applied to the *p*-values from the pairwise comparisons, and the adjusted significance threshold was set accordingly (e.g., *p* < 0.005 when comparing all five groups).

## 3. Results

### 3.1. Preparation and Characterization of Green-Synthesized NSF, Structural and Morphological Analysis

**Figure 1:**
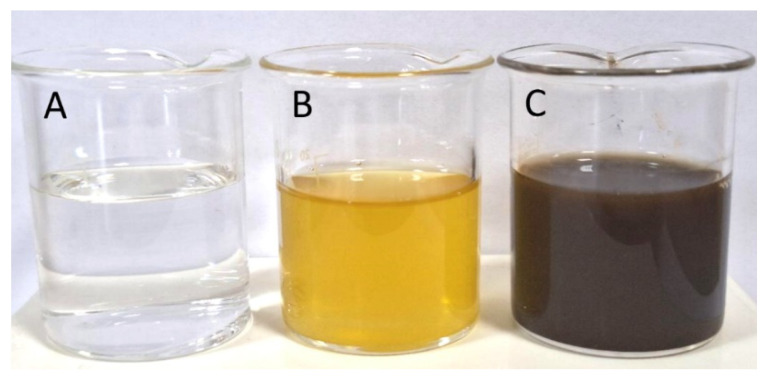
Image of (**A**)—aqueous solution of silver nitrate; (**B**)—water extract of *Camellia sinensis* green tea leaves; (**C**)—suspension of the formulated silver nanoparticles.

The reduction of silver ions in the synthesis of nanoparticles was confirmed visually by the change in the color of the reaction medium. Within less than one hour after mixing the silver nitrate solution and the green tea extract, the color of the system changed from yellow to dark brown, indicating the formation of silver nanostructures (Figure 1).

**Figure 2:**
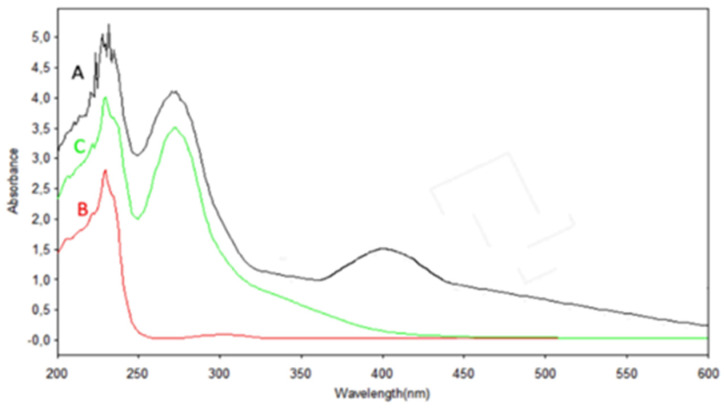
UV-Vis spectra of (**A**)—suspension of the formulated silver nanoparticles; (**B**)—silver nitrate aqueous solution; (**C**)—aqueous extract of green tea leaves *Camellia sinensis*.

Figure 2 presents the UV-Vis absorption spectra of the reagents used in the synthesis of the nanoparticles as well as the spectrum of the resulting suspension of silver nanostructures after appropriate dilution with purified water. The silver nitrate solution has no absorption in the range of 250 to 600 nm (Figure 2B), while green tea extract has an absorption maximum at a wavelength of 273–277 nm (Figure 2C) due to the organic compounds present in it. The pronounced surface plasmon resonance at 400 nm in the spectrum of the nanostructures suspension (Figure 2A) indicates the formation of spherical silver nanoparticles.

The silver fluoride nanoparticles were characterized in terms of average particle diameter and particle size distribution by dynamic light scattering. Their shape, morphology, and elemental composition were analyzed using transmission and scanning electron microscopy.

**Figure 3:**
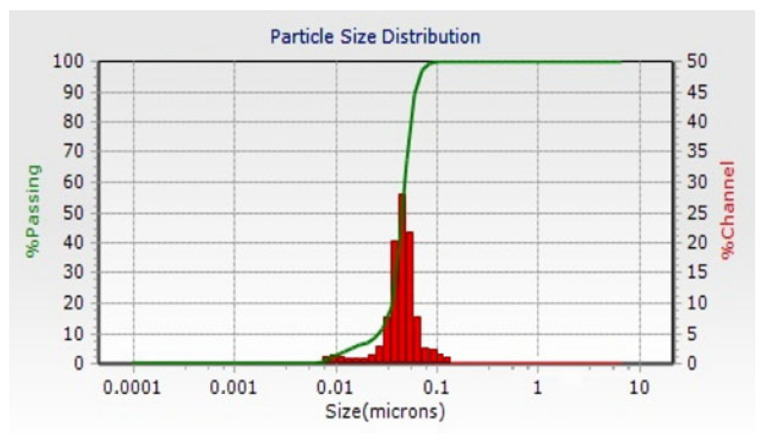
DLS histogram of silver nanoparticles.

The particle size distribution of the resulting nanoparticles is presented in Figure 3. Narrow monomodal size distributed was observed, mainly in the range from 10 to 100 nm. The average diameter of the nanoparticles, determined based on three measurements, was 65 nm (±14 SD).

**Figure 4:**
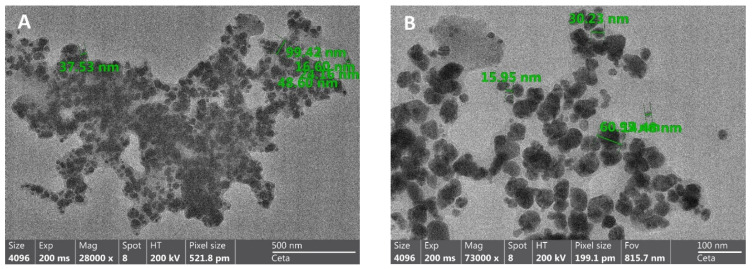
TEM micrographs of silver nanoparticles at a magnification of 28,000× (**A**) and at a magnification of 73,000× (**B**).

Transmission electron microscopy was used to visualize the formulated nanoparticles. Figure 4 presents micrographs with different magnification of the resulting particles. The formed structures have a relatively uniform size and size distribution below 100 nm.

**Figure 5:**
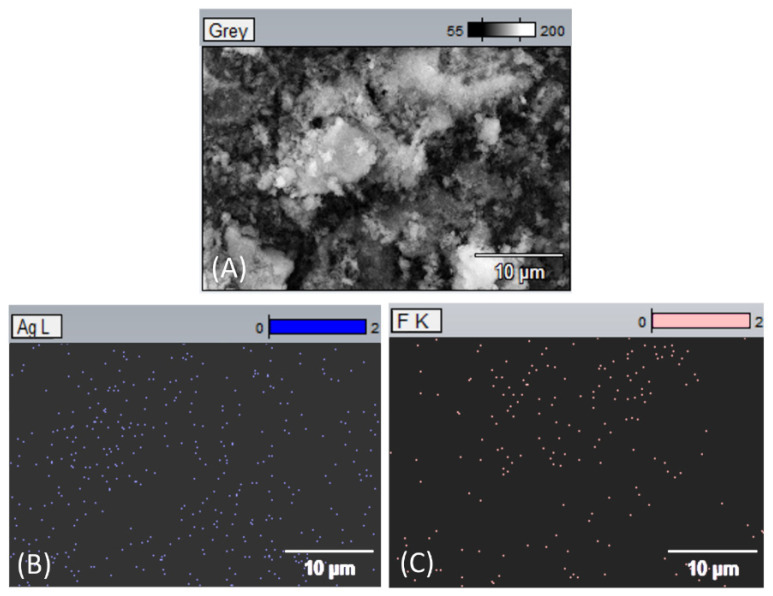
EDX-elemental analysis of nanoparticles: SEM micrograph of lyophilized particles (**A**); presence of silver (**B**) and fluoride (**C**) in the observed sample.

Figure 5 presents a micrograph of lyophilized nanoparticles obtained by scanning electron microscopy. An EDX analysis of the surface elemental composition of the observed sample was performed at different magnifications. It was clearly seen that besides silver (Figure 5B), incorporated fluoride was also found in the formulated silver nanoparticles (Figure 5C).

### 3.2. Surface Microhardness Assessment

The newly defined variables (VHN-1 and VHN-2) did not follow a normal distribution (Shapiro-Wilk—*p* < 0.05). The Kruskal-Wallis test showed a statistically significant difference between the studied groups (*p* < 0.05). To identify the specific groups between which there are statistically significant differences, the Mann–Whitney U test with Bonferroni corrections was used.

Table 3 summarizes the medians (Median) and interquartile ranges (IQR), as well as the results of the comparative statistical analysis between the studied groups after the definition of the two new variables (VHN-1 and VHN-2).

The variable VHN-1 (VHN-C minus VHN-S) had negative values in all five groups, indicating a decrease in the specimen’s microhardness after demineralization and formation of initial carious lesions. The variable VHN-2 (VHN-T minus VHN-C) had positive values in all groups, which was indicative of remineralization. The highest median of VHN-2 was observed in Gr-NSFd (29.15, IQR = 14.78), which proves the best remineralization potential in this group. The second highest median was observed in Gr-RS (27.60, IQR = 50.83), and the differences in VHN-2 between Gr-NSFd and Gr-RS were not statistically significant (*p* = 1.000). From the results obtained, it can be concluded that these two groups have a similar remineralization effect. The lowest median was observed in the control group Gr-C (2.85, IQR = 7.80), whose samples were not treated with a silver compound but only underwent pH cycling.

**Figure 6:**
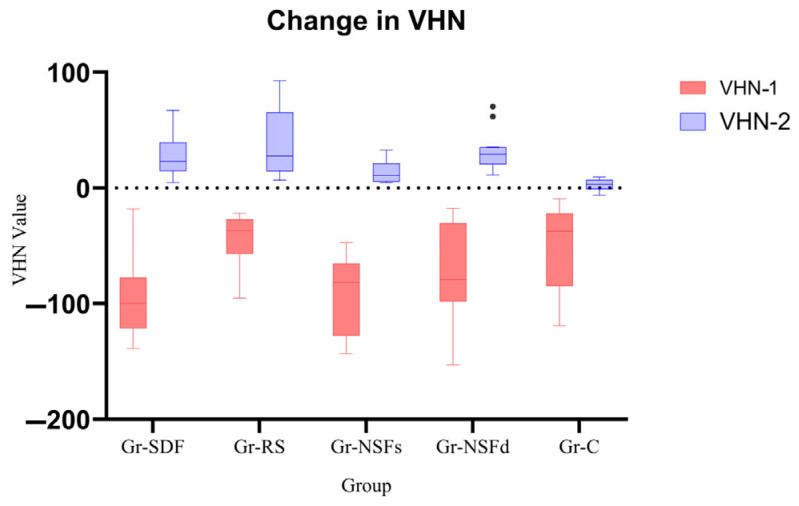
Change in VHN-1 and VHN-2 amongst studied groups.

Based on the results, the experimental groups can be arranged according to the remineralizing potential of the material used from highest to lowest effect in the following order: Gr-NSFd, Gr-RS, Gr-SDF, Gr-NSFs, Gr-C (Figure 6).

### 3.3. Photographic Analysis

**Figure 7:**
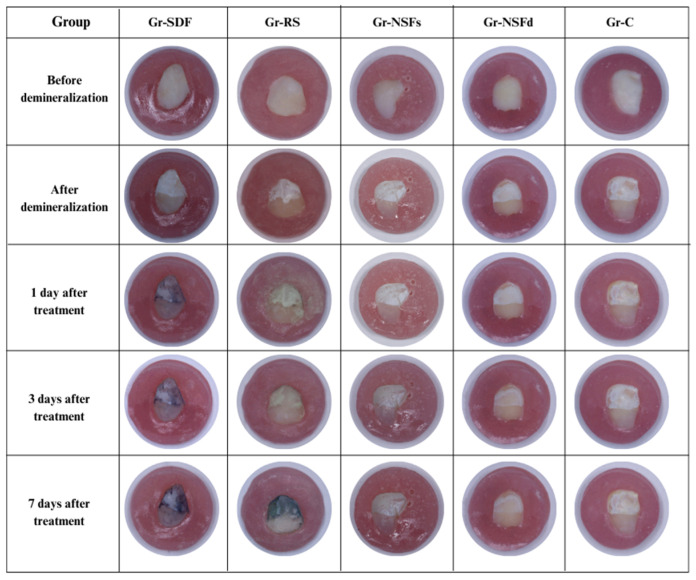
Photographic analysis of the studied groups.

Photographic analysis from each studied group is presented in Figure 7. In all groups, the samples before demineralization had intact smooth enamel surface, without visible roughness, cracks, structural defects, or staining. After demineralization, white spots were observed in the experimental window, which visually indicate the formed initial carious lesions. In Gr-SDF, a distinct black staining of the samples was observed on the first day after application of 38% SDF. In Gr-RS, in the first days after treatment with 38% SDF/KI, there was no discoloration of the teeth, but on the 7th day, such was already observed. In contrast to these groups, in the samples treated with NSF (Gr-NSFs and Gr-NSFd) at all points of the photographic analysis, no change in tooth color was observed. The control group Gr-C, which lacked treatment with silver compounds, also showed no signs of staining. This confirms that the solutions used for pH cycling do not contribute to additional staining of the tooth structures, which excludes the possibility that they are the cause of the reported color changes in some of the experimental groups.

## 4. Discussion

Based on the results and statistical findings, the null hypothesis was rejected.

The main technological parameters that affect the described method for green synthesis of silver nanoparticles and their sizes are temperature, pH of the medium, concentration of the silver nitrate solution, and amount of green tea extract used. According to literature data, increasing the temperature of the reaction system within certain limits has a positive effect on the yield of silver nanoparticles [38]. This can be explained by the formation of a larger number of new silver particles at a higher temperature, at the expense of the growth of already formed silver crystals [39]. On the other hand, too high temperatures pose a risk to the stability of the formed structures. It has been established that a temperature of 80 °C is optimal for the biosynthesis of silver nanoparticles [40]. pH is another important factor affecting the yield and size of the synthesized nanostructures [41]. Hunag et al. found that the preparation of silver nanoparticles by a green synthesis method was most effective when the medium was alkalized to pH 9. Above this value, the formation of nanostructures was delayed, and the tendency to aggregation was increased [40]. The 1:1 ratio between the aqueous extract of green tea and the AgNO_3_ led to the formation of nanoparticles below 100 nm.

TEM analysis confirmed that the formed nanoparticles have a compact spherical morphology and dimensions below 100 nm, which is the desired characteristic for potential biomedical applications. EDX analysis demonstrated that in addition to silver, fluoride is also present in the nanoparticles, suggesting possible additional bioactivity of the obtained material.

To determine which silver compound demonstrates the best remineralizing effect on incipient enamel lesions, the study design included five study groups—four groups treated with different silver agents and application rates, and one control group with no treatment. After application of the respective material, each of the experimental groups underwent chemical pH cycling. This method allowed the dynamics of demineralization-remineralization processes to be reproduced in laboratory conditions [42]. However, this methodology cannot fully recreate natural intraoral conditions, as it does not consider key factors such as salivary flow rate, ionic composition, pH, and buffering capacity of saliva [43,44]. Furthermore, the chemical pH cycling model does not include the impact of bacterial biofilm—an essential component of the cariogenic environment [45]. Due to these limitations, in the present study, the effect of the silver compounds investigated was evaluated solely based on their remineralizing properties in vitro.

Another limitation of this study is that full blinding could not be ensured during outcome assessment due to the visible black staining in some treatment groups. Although samples were coded and assessments were performed by an independent examiner, the nature of the discoloration made group identification unavoidable.

Evidence regarding the effect of silver compounds on incipient enamel lesions in primary dentition is still limited. Traditionally, their main application was related to managing dentinal carious lesions, where they have been shown to arrest the demineralization process. Although their effect on enamel lesions is less well studied, the available data suggest that they may have significant potential to stimulate remineralization and serve as an adjunct therapy to other dental interventions [46].

Several in vitro studies have investigated the remineralizing potential of 38% SDF in the treatment of incipient carious lesions, with the main indicator of its effectiveness being the change in surface microhardness [14,47,48]. Despite the proven remineralization effect, one of the main limitations is related to the significant black staining of dental tissues after application.

The use of 38% SDF with KI for the treatment of early enamel lesions has been studied to a very limited extent. However, the available literature data indicate that the combination of these materials successfully remineralizes white spot lesions. Lee et al. demonstrated that 38% SDF/KI is effective in remineralizing tooth enamel, and the application of KI reduces the degree of tooth staining [16].

NSF is a relatively new development and alternative to the previously known silver compounds, possessing remineralizing properties without staining the teeth black. As far as is known, no studies have been reported to date on the microhardness of enamel samples treated with green-synthesized NSF. The available literature data examine other formulations of nano-silver particles, synthesized by chemical methods and with different methodologies, which also affect their healing effect. Nozari et al. investigated the remineralization potential of chemically prepared NSF compared to 5% sodium fluoride (NaF) varnish and nano-hydroxyapatite serum on artificial white spot lesions in primary teeth. They concluded that all products have similar remineralization features [25]. Silva et al. used NSF, synthesized with chitosan as a carrier and stabilizer, and proved that it has greater remineralization features in comparison with NaF [49]. In a very recent study, Albahoth et al. used L-arginine to produce NSF and proved that it could be a suitable substitute for SDF for remineralizing incipient enamel lesions in primary teeth [50]. The promising outcomes from the in vitro studies including nano-silver particles provide a rationale for conducting clinical trials with similar materials. Moreover, emerging evidence from existing clinical trials has already demonstrated promising outcomes, highlighting their potential applicability in the clinical setting. For example, Quritum et al. [51] compared the effect of a novel, chitosan-stabilized NSF and 38% SDF among 360 children under the age of 4. Their study demonstrated that NSF successfully arrests caries lesions without staining the teeth in black and shows better parent satisfaction, making it a suitable alternative to SDF. Atteya et al. [52] investigated the effect of newly developed NSF varnish on incipient enamel lesions. Its application showed significant reduction in caries lesion activity, which was comparable to traditionally used NaF varnish. Despite these encouraging findings, there is still a lack of well-designed clinical trials specifically evaluating green-synthesized NSF, which limits the ability to translate current in vitro findings to clinical settings.

Additionally, other compounds such as casein phosphopeptide-amorphous calcium phosphate [53] and biomimetic hydroxyapatite [54] showed promising results, so their supplementation in combination with NSF could be explored in future studies to enhance therapeutic outcomes.

Building on these findings from chemically synthesized NSF, the present study provides new insight into the effectiveness of green-synthesized NSF, which also offers the added advantage of being sustainable and eco-friendly.

## 5. Conclusions

The present results prove that the use of green tea as a reducing agent is an effective and environmentally safe method for the synthesis of silver nanoparticles. The optimized reaction conditions allow the preparation of particles with the desired sizes and morphology.

The obtained results of the microhardness change of enamel samples show promising trends. Two applications of NSF within a one-week period demonstrate better remineralization effect compared to a single application. The remineralizing effect of twice applied NSF is comparable to a single application of SDF/KI, with no significant difference between them. A serious advantage of the proposed method of green-synthesized NFS is that it does not cause color changes in the experimental samples, which makes it more favorable for clinical application, especially in aesthetically important areas. Additional studies are required to fully establish the efficacy, safety, clinical applicability, and long-term effect of green-synthesized NSF in pediatric dentistry.

## Figures and Tables

**Table 1:** Materials and application protocol.

Group	Material	Composition	Application Protocol
Gr-SDF	38% SDF (Riva Star Step 1, SDI Dental Limited, Melbourne, Victoria, Australia)	Silver (Ag), fluoride (F), ammonia (NH_3_)	One drop of Step 1 capsule is applied with a microbrush for 60 s. Excess material is removed using sterile gauze.
Gr-RS	38% SDF + KI (Riva Star Step 1 + Step 2, SDI Dental Limited, Melbourne, Victoria, Australia)	Step 1: Silver (Ag), fluoride (F), ammonia (NH_3_) Step 2: potassium iodide (KI)	One drop of Step 1 capsule is applied with a microbrush for 60 s. Afterwards, two drops of Step 2 capsule are applied, resulting in a milky-white precipitate. Application continues until the precipitation becomes transparent. Excess material is removed using sterile gauze.
Gr-NSFs	Green-synthesized NSF (laboratory prepared)	AgNO_3_, K_2_CO_3_, NaF, aqueous extract of *Camellia sinensis*	The solution is applied with a microbrush for 60 s. Excess material is removed with sterile gauze.
Gr-NSFd	Green-synthesized NSF (laboratory prepared)	AgNO_3_, K_2_CO_3_, NaF, aqueous extract of *Camellia sinensis*;	Same protocol as for Gr-NSFs, repeated twice at a one-week interval.
Gr-C	Control (no treatment)	-	No treatment applied. Underwent pH cycling only.

**Table 2:** Composition of de- and remineralizing solutions.

Solution Type	Composition	pH
Demineralizing	CaCl_2_, NaH_2_PO_4_, CH_3_COOH, KOH	4.4
Remineralizing	NaCl, C_3_H_6_O_3_, NaOH	7.4 ± 0.1

**Table 3:** Results from Kruskal-Wallis and Mann–Whitney U tests.

	Gr-SDF (1)	Gr-RS (2)	Gr-NSFs (3)	Gr-NSFd (4)	Gr-C (5)	*p*-Value M–W U Test
Variable	Median (IQR)	Median (IQR)	Median (IQR)	Median (IQR)	Median (IQR)	
VHN-1	−99.85 (43.88)	−37.00 (29.75)	−81.95 (62.30)	−79.15 (67.85)	−37.65 (62.83)	2↔3: *p* < 0.001
VHN-2	23.10 (24.88)	27.60 (50.83)	10.65 (15.50)	29.15 (14.78)	2.85 (7.80)	1↔5: *p* < 0.001 2↔5: *p* < 0.001 4↔5: *p* < 0.001

## Data Availability

The data supporting the findings of this study are available from the corresponding author upon reasonable request.

## Abbreviations

The following abbreviations are used in this manuscript:

ECC	early childhood caries
SDF	silver diamine fluoride
KI	potassium iodide
NSF	nano-silver fluoride
TEM	transmission electron microscopy
SEM	scanning electron microscopy
DLS	dynamic light scattering
EDX	energy-dispersive X-ray
VHN	Vicker’s hardness number
IQR	interquartile ranges
NaF	sodium fluoride
